# Supplementary figures and images for: NOLC1 suppresses immunochemotherapy by inhibiting p53-mediated ferroptosis in gastric cancer
Source: eLife. 2025 Aug 27;13:RP103904. doi: 10.7554/eLife.103904 (PMC12387760; doi:10.7554/eLife.103904)

Fig.1E

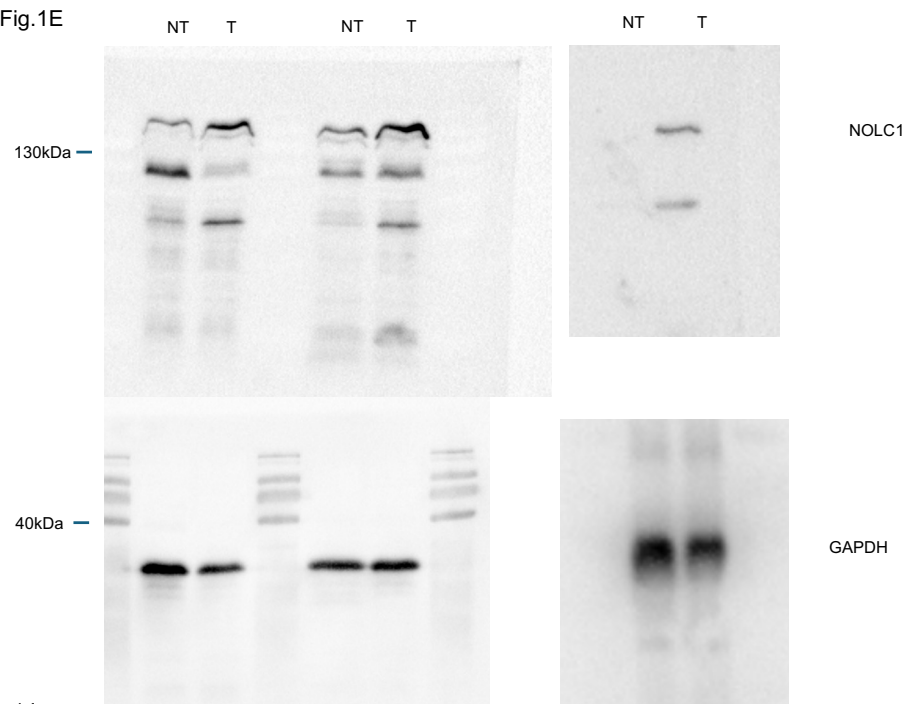

Fig.1J

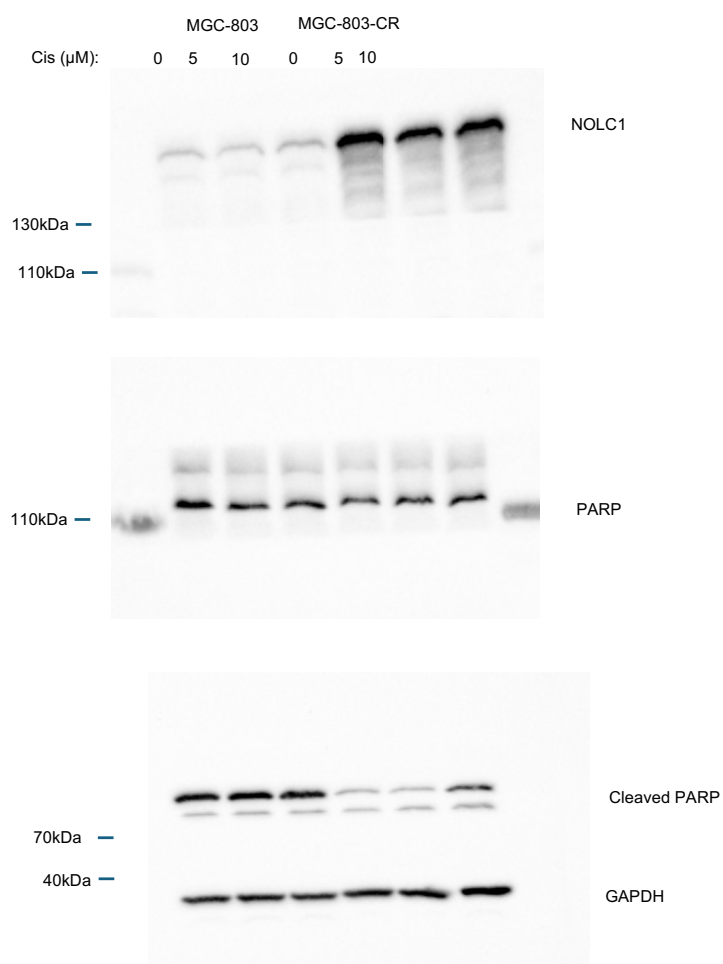

Supplement: Figure 1—source data 1. [file elife-103904-fig1-data1.zip › fig1-source data1.pdf]

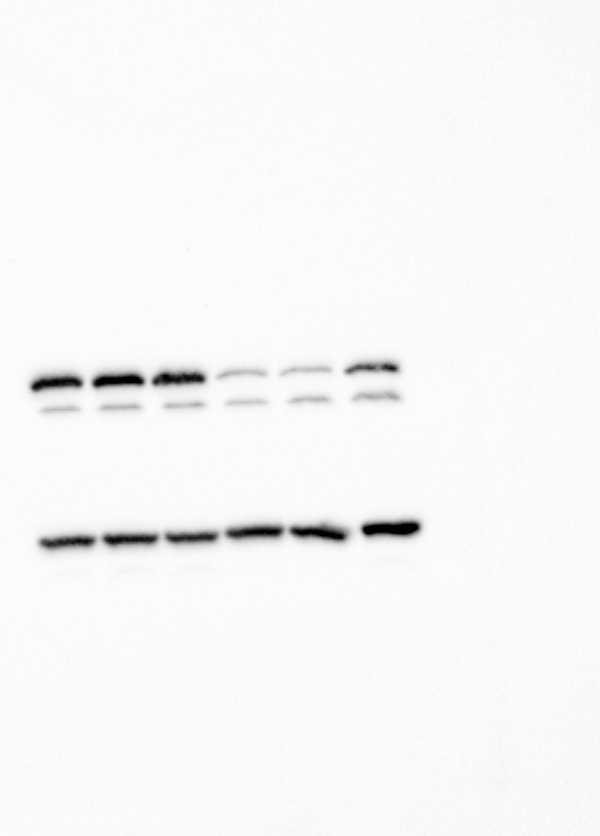

Supplement: Figure 1—source data 2. [file elife-103904-fig1-data2.zip › fig1 source data2/FIG1J/CR-CL-parp gap.2.jpg]

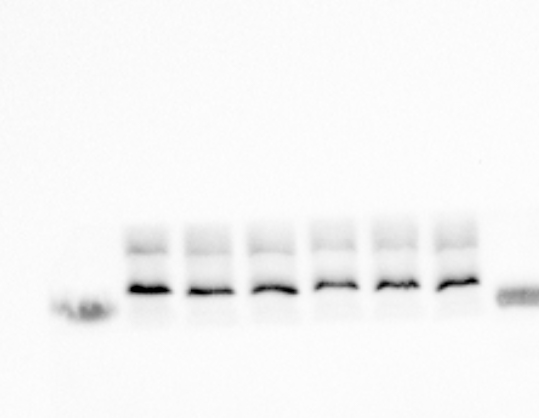

Supplement: Figure 1—source data 2. [file elife-103904-fig1-data2.zip › fig1 source data2/FIG1J/GAP parp.jpg]

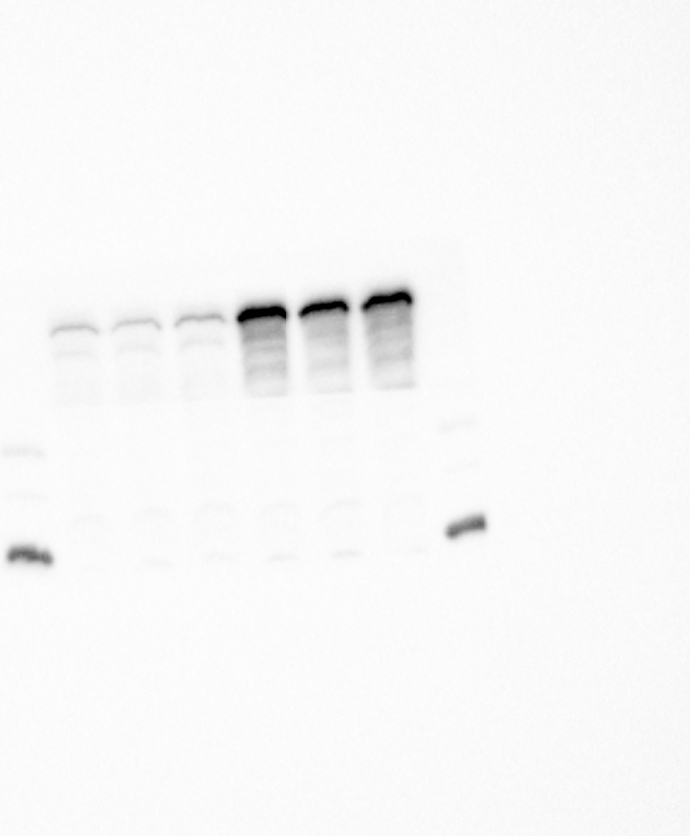

Supplement: Figure 1—source data 2. [file elife-103904-fig1-data2.zip › fig1 source data2/FIG1J/NOLC1.1.jpg]

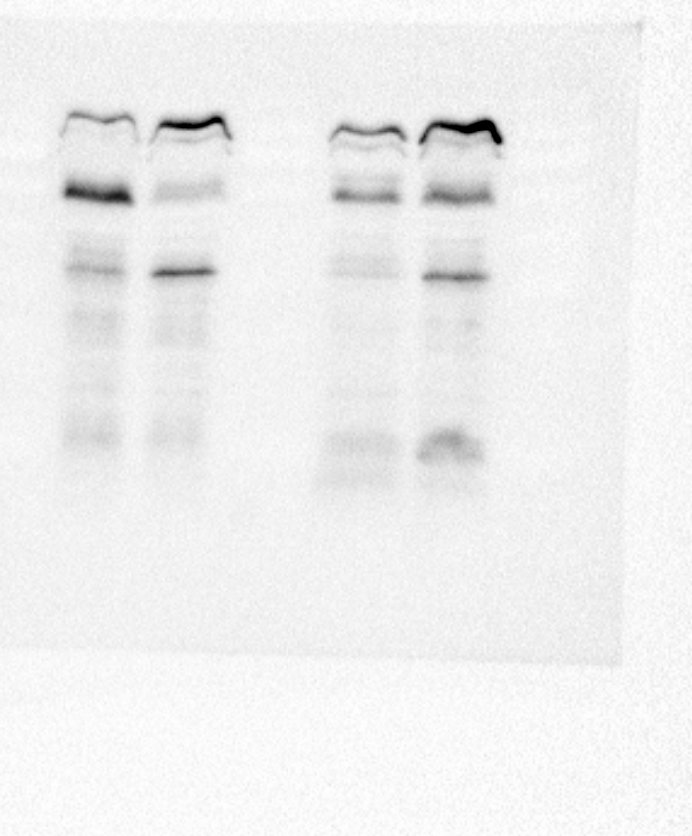

Supplement: Figure 1—source data 2. [file elife-103904-fig1-data2.zip › fig1 source data2/FIG1E/Fig1E NOLC1.jpg]

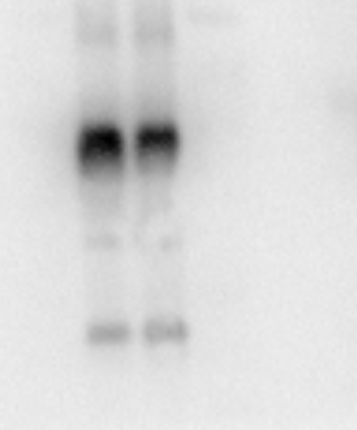

Supplement: Figure 1—source data 2. [file elife-103904-fig1-data2.zip › fig1 source data2/FIG1E/FIG1E-P3-GAPDH.jpg]

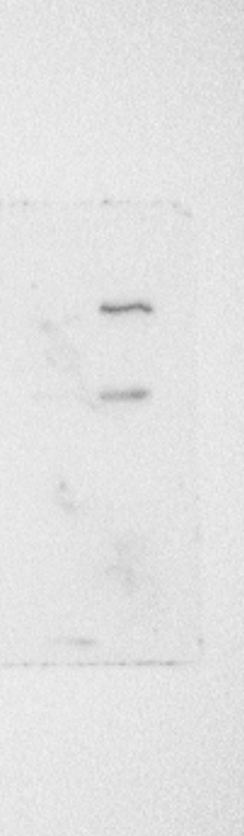

Supplement: Figure 1—source data 2. [file elife-103904-fig1-data2.zip › fig1 source data2/FIG1E/P3-NOLC1.jpg]

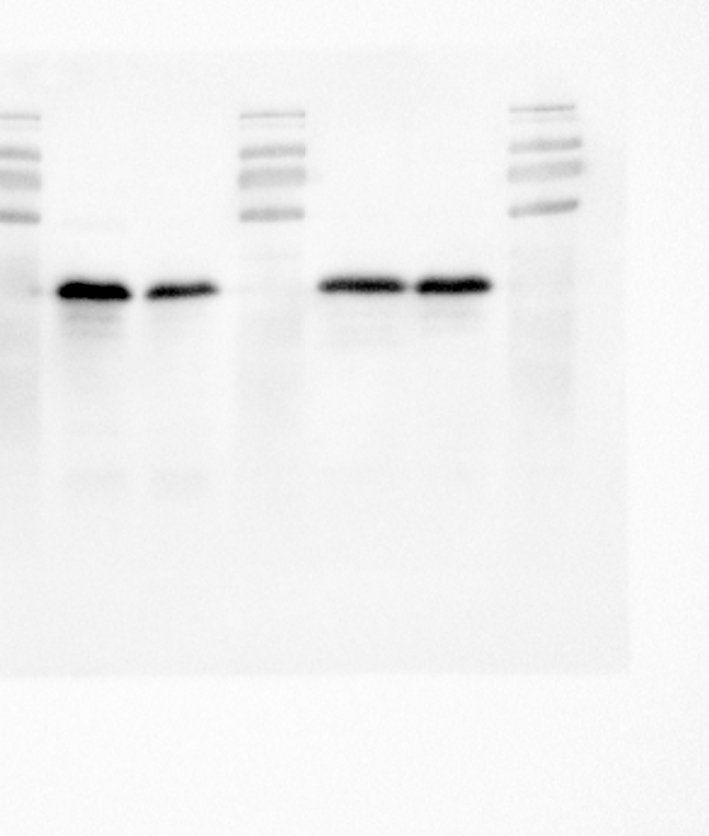

Supplement: Figure 1—source data 2. [file elife-103904-fig1-data2.zip › fig1 source data2/FIG1E/Fig1E GAPDH.jpg]

Fig.2J

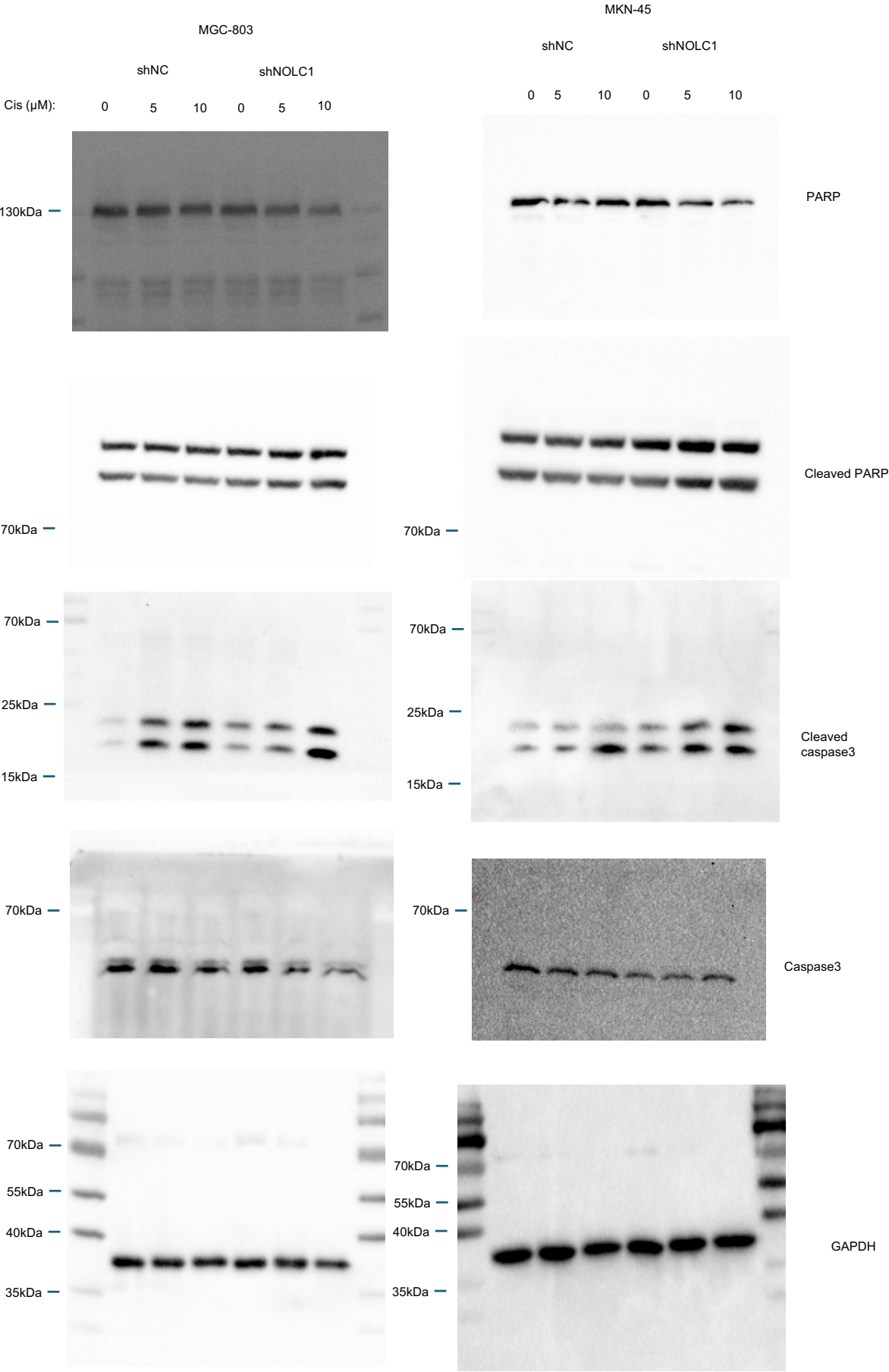

Supplement: Figure 2—source data 1. [file elife-103904-fig2-data1.pdf]

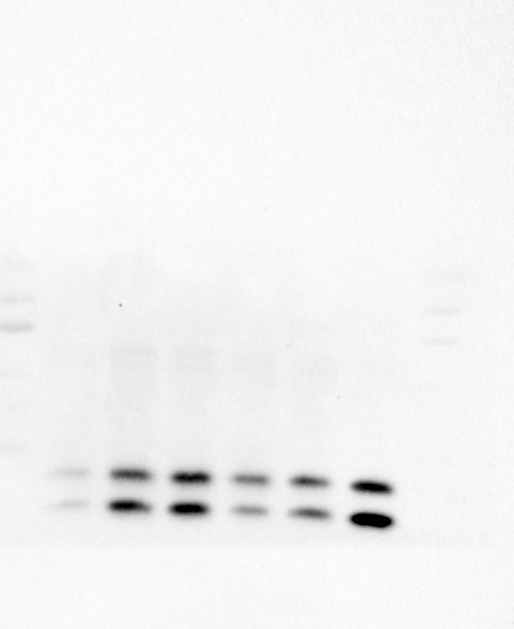

Supplement: Figure 2—source data 2. [file elife-103904-fig2-data2.zip › fig2 source data2/FIG2J/MGC-clcaspase3.jpg]

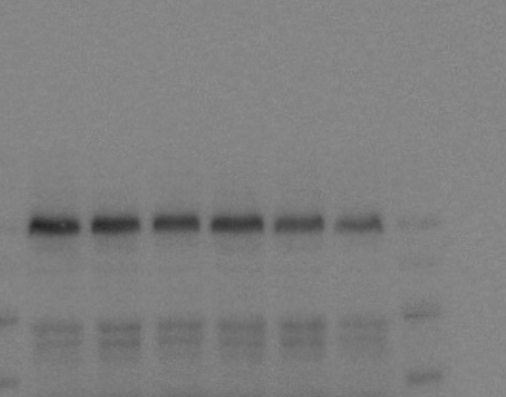

Supplement: Figure 2—source data 2. [file elife-103904-fig2-data2.zip › fig2 source data2/FIG2J/MGC-PARP.jpg]

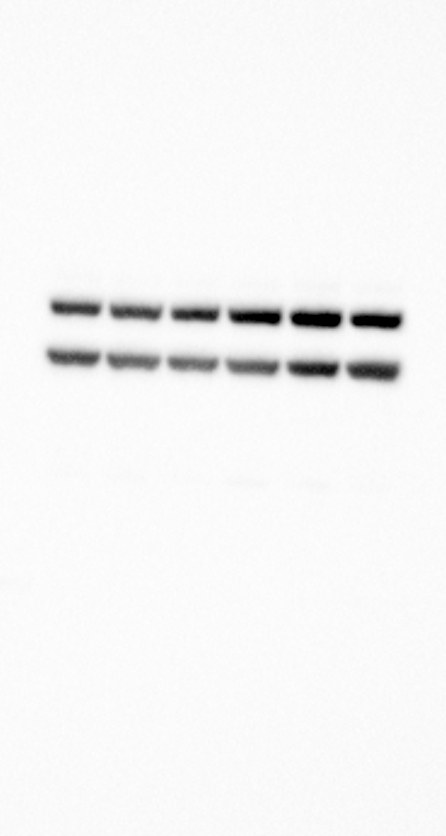

Supplement: Figure 2—source data 2. [file elife-103904-fig2-data2.zip › fig2 source data2/FIG2J/MKN-cl-parp.jpg]

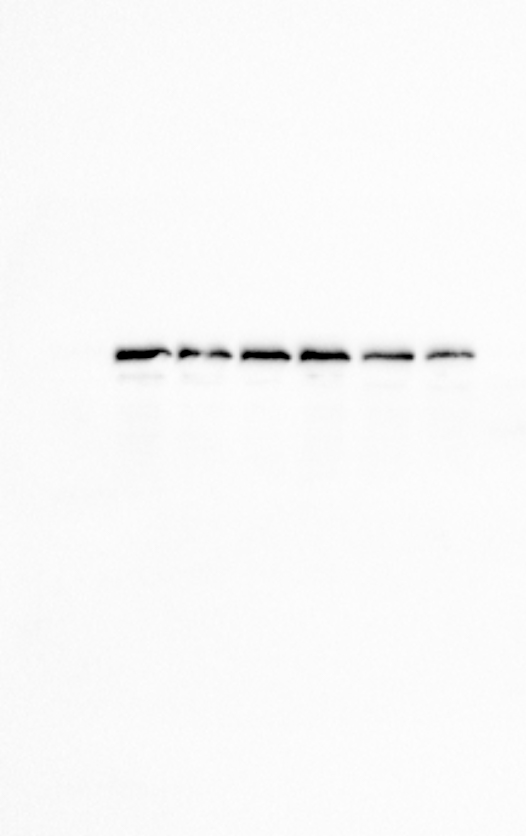

Supplement: Figure 2—source data 2. [file elife-103904-fig2-data2.zip › fig2 source data2/FIG2J/MKN-PARP.jpg]

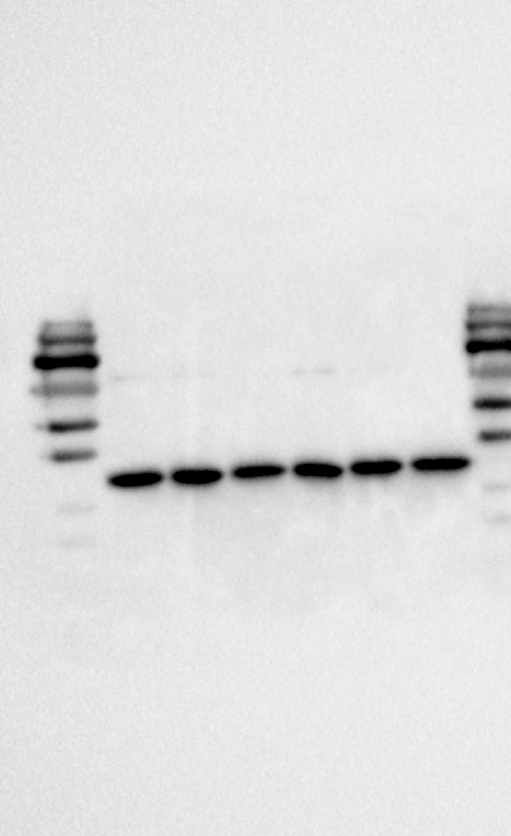

Supplement: Figure 2—source data 2. [file elife-103904-fig2-data2.zip › fig2 source data2/FIG2J/MKN-GAP.jpg]

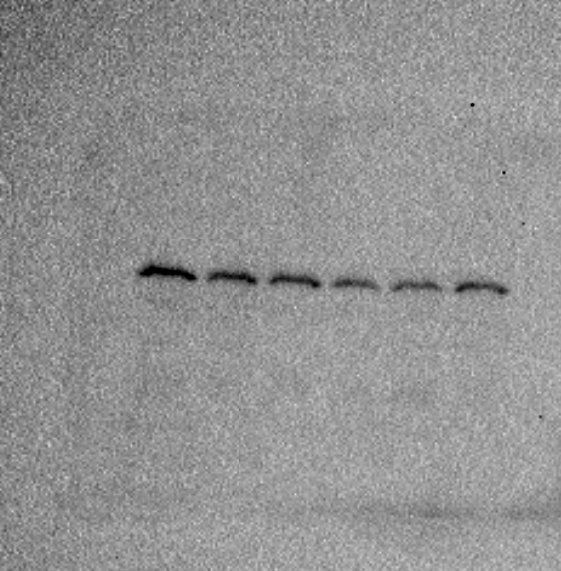

Supplement: Figure 2—source data 2. [file elife-103904-fig2-data2.zip › fig2 source data2/FIG2J/MKN-Caspase3.jpg]

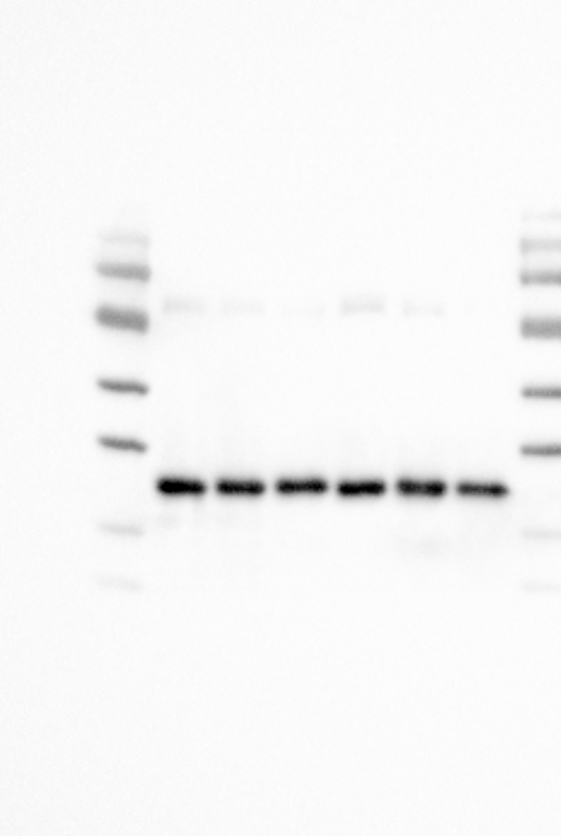

Supplement: Figure 2—source data 2. [file elife-103904-fig2-data2.zip › fig2 source data2/FIG2J/MGC-GAP.jpg]

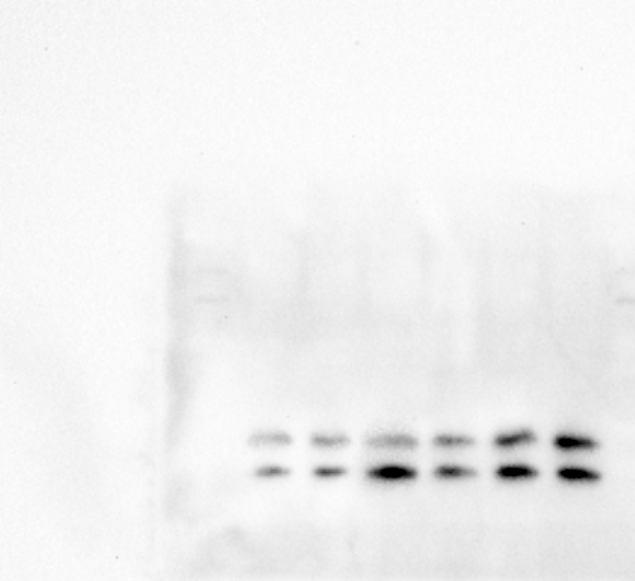

Supplement: Figure 2—source data 2. [file elife-103904-fig2-data2.zip › fig2 source data2/FIG2J/MKN-CL-CASPASE3-3.jpg]

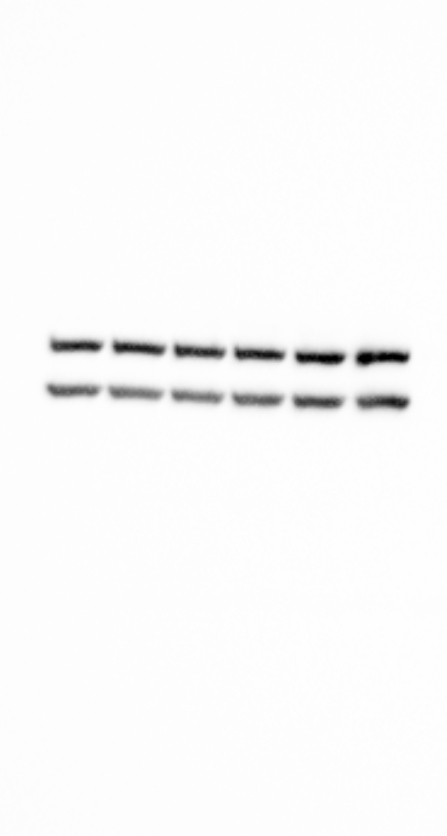

Supplement: Figure 2—source data 2. [file elife-103904-fig2-data2.zip › fig2 source data2/FIG2J/MGC-CL-PARP.jpg]

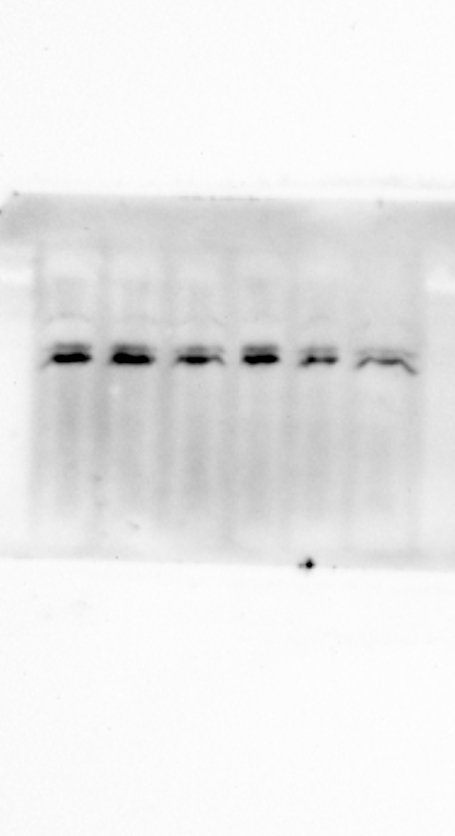

Supplement: Figure 2—source data 2. [file elife-103904-fig2-data2.zip › fig2 source data2/FIG2J/MGC-CAS3.jpg]

**Figure 2-figure supplement 1C**

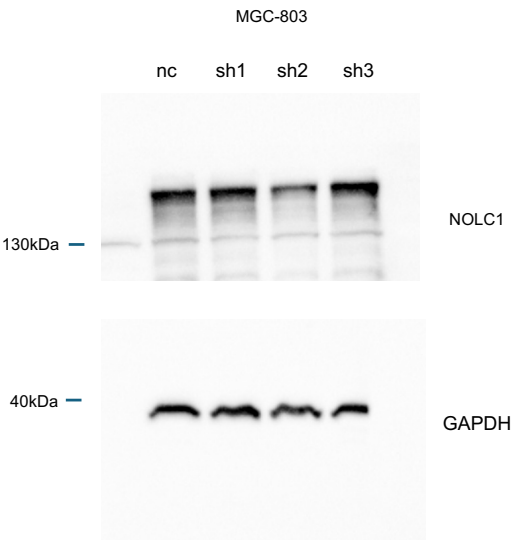

**Figure 2-figure supplement 1D**

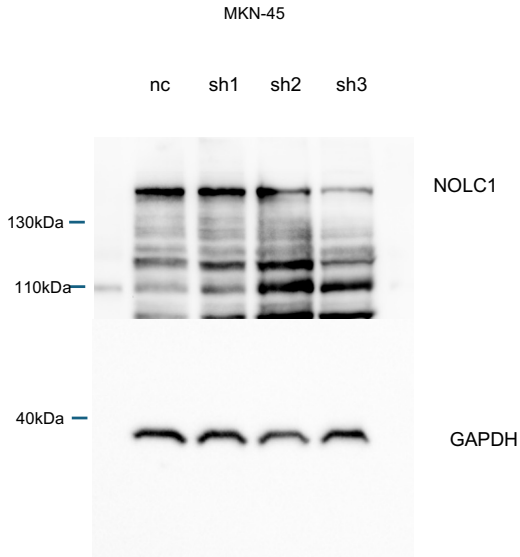

**Figure 2-figure supplement 1E**

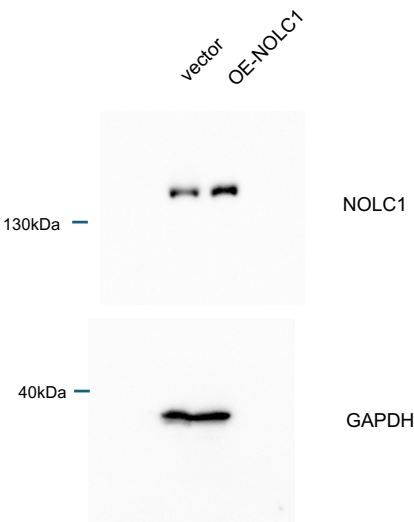

Supplement: Figure 2—figure supplement 1—source data 1. [file elife-103904-fig2-figsupp1-data1.pdf]

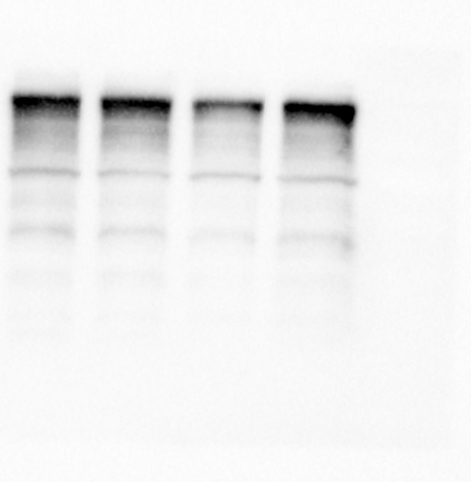

Supplement: Figure 2—figure supplement 1—source data 2. [file elife-103904-fig2-figsupp1-data2.zip › fig2 supplement1 source data2/si-NOLC1-MGC.jpg]

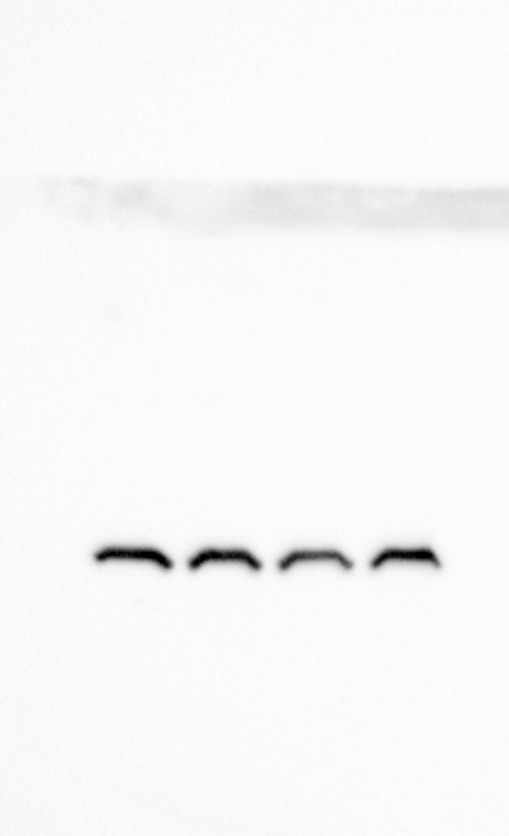

Supplement: Figure 2—figure supplement 1—source data 2. [file elife-103904-fig2-figsupp1-data2.zip › fig2 supplement1 source data2/si-NOLC1-MKN-GAPDH.jpg]

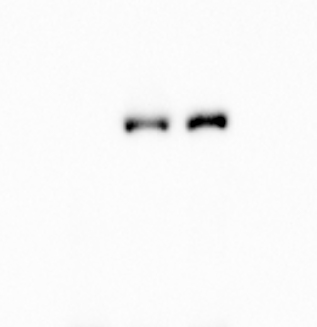

Supplement: Figure 2—figure supplement 1—source data 2. [file elife-103904-fig2-figsupp1-data2.zip › fig2 supplement1 source data2/OE-NOLC1.jpg]

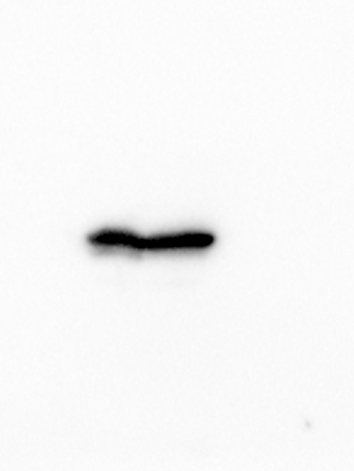

Supplement: Figure 2—figure supplement 1—source data 2. [file elife-103904-fig2-figsupp1-data2.zip › fig2 supplement1 source data2/GAP-OE.jpg]

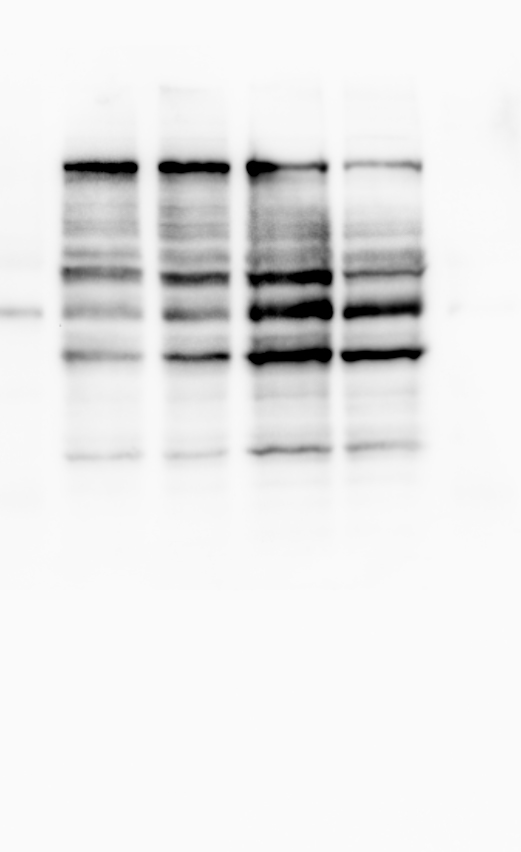

Supplement: Figure 2—figure supplement 1—source data 2. [file elife-103904-fig2-figsupp1-data2.zip › fig2 supplement1 source data2/si-NOLC1-MKN.jpg]

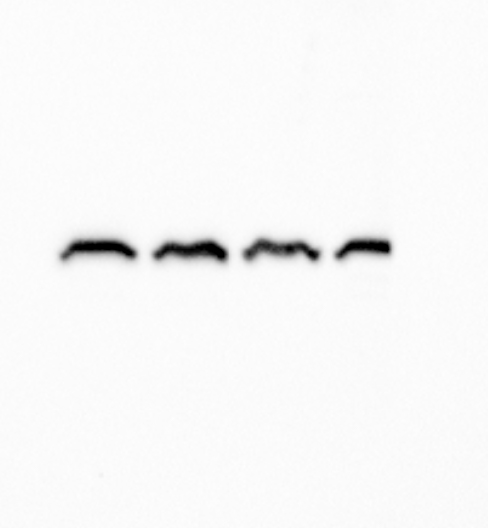

Supplement: Figure 2—figure supplement 1—source data 2. [file elife-103904-fig2-figsupp1-data2.zip › fig2 supplement1 source data2/si-NOLC1-MGC-GAPDH.jpg]

Fig.4H

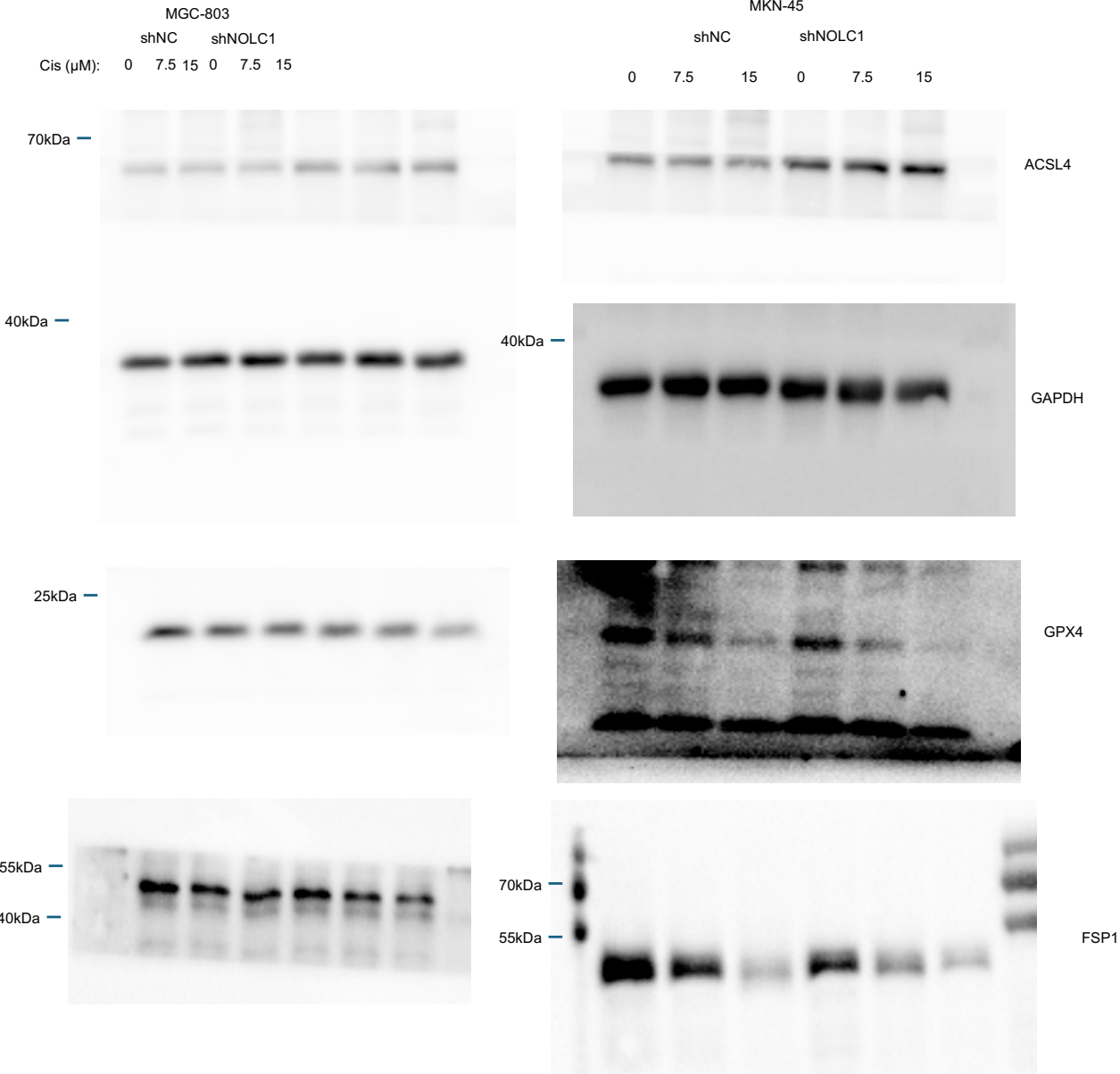

Supplement: Figure 4—source data 1. [file elife-103904-fig4-data1.pdf]

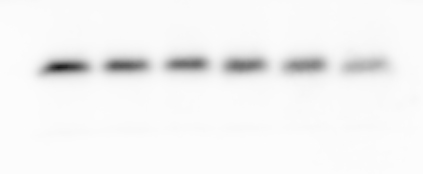

Supplement: Figure 4—source data 2. [file elife-103904-fig4-data2.zip › fig4 source data2/fig.4h/ MGC-GPX4.jpg]

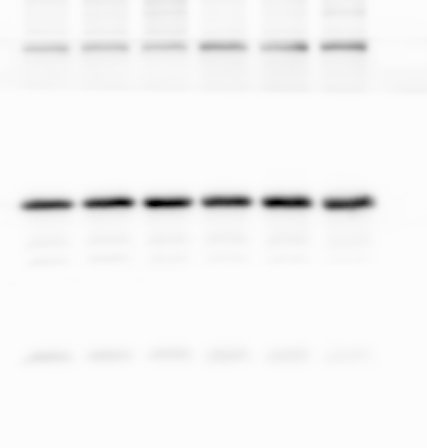

Supplement: Figure 4—source data 2. [file elife-103904-fig4-data2.zip › fig4 source data2/fig.4h/MGC-ACSL4-GAP.jpg]

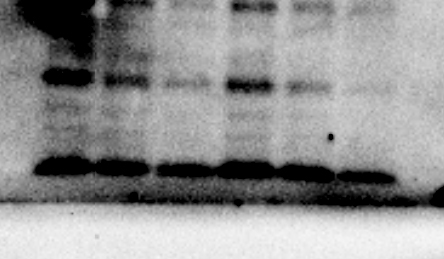

Supplement: Figure 4—source data 2. [file elife-103904-fig4-data2.zip › fig4 source data2/fig.4h/MKN-GPX4.jpg]

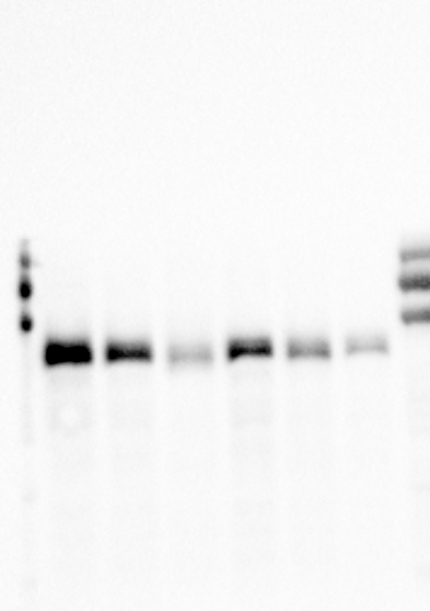

Supplement: Figure 4—source data 2. [file elife-103904-fig4-data2.zip › fig4 source data2/fig.4h/MKN-FSP.jpg]

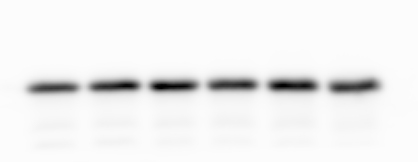

Supplement: Figure 4—source data 2. [file elife-103904-fig4-data2.zip › fig4 source data2/fig.4h/MGC-GAPDH_.jpg]

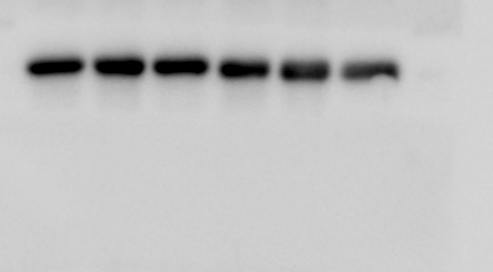

Supplement: Figure 4—source data 2. [file elife-103904-fig4-data2.zip › fig4 source data2/fig.4h/MKN-GAP.jpg]

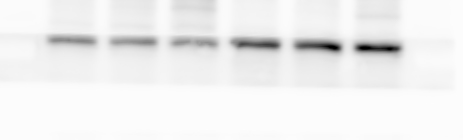

Supplement: Figure 4—source data 2. [file elife-103904-fig4-data2.zip › fig4 source data2/fig.4h/MKN-ACSL4jpg.jpg]

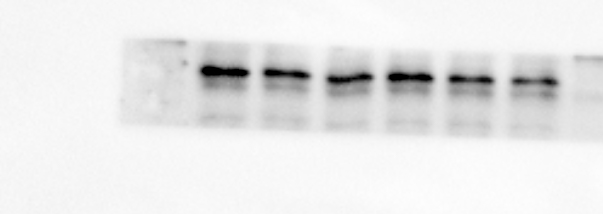

Supplement: Figure 4—source data 2. [file elife-103904-fig4-data2.zip › fig4 source data2/fig.4h/MGC-FSP.jpg]

Fig.5A

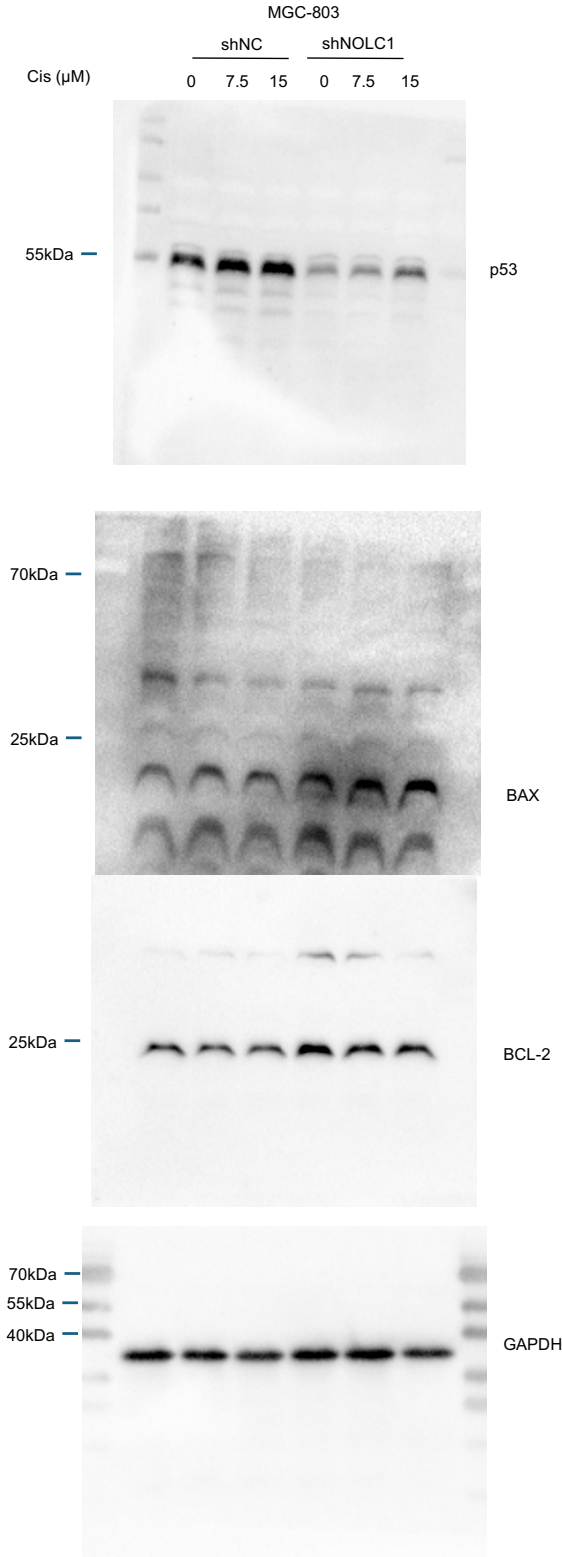

Fig.5G

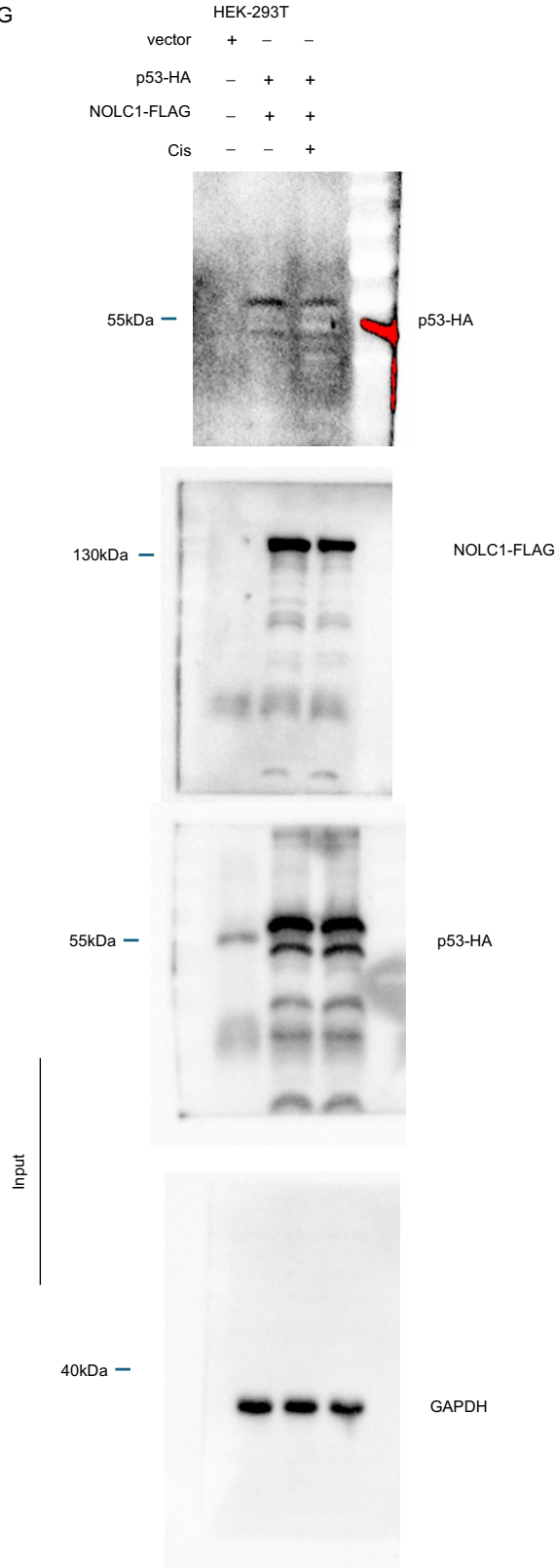

Fig.5I

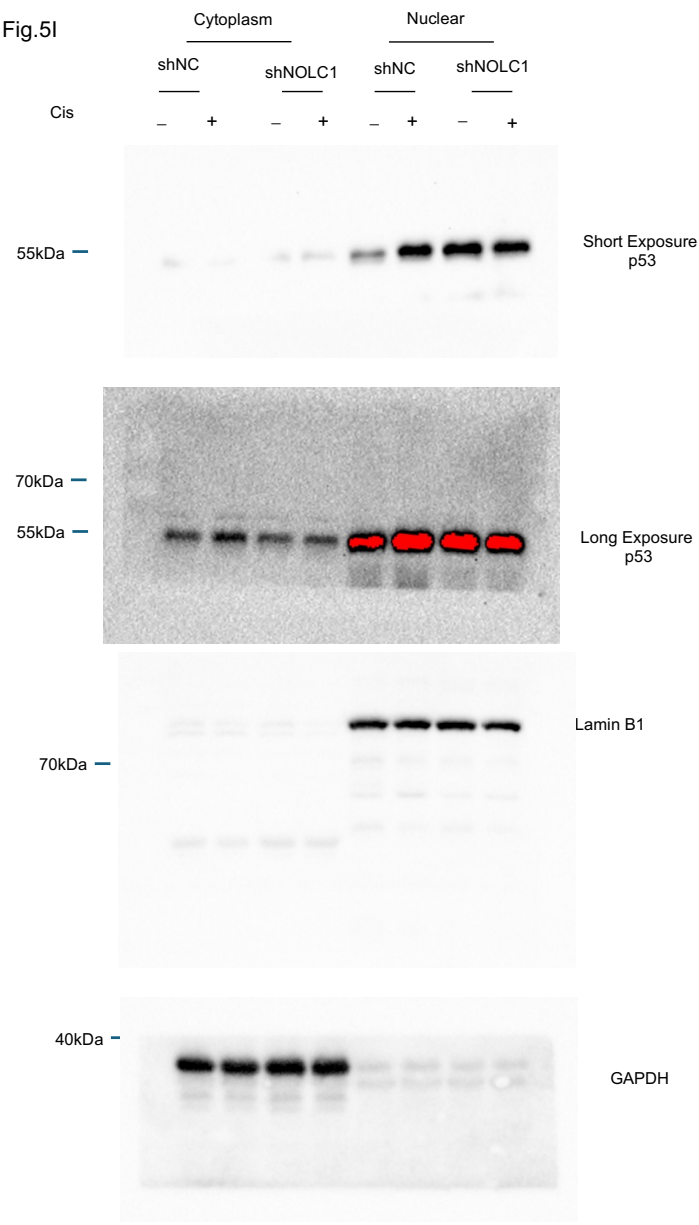

Fig.5K

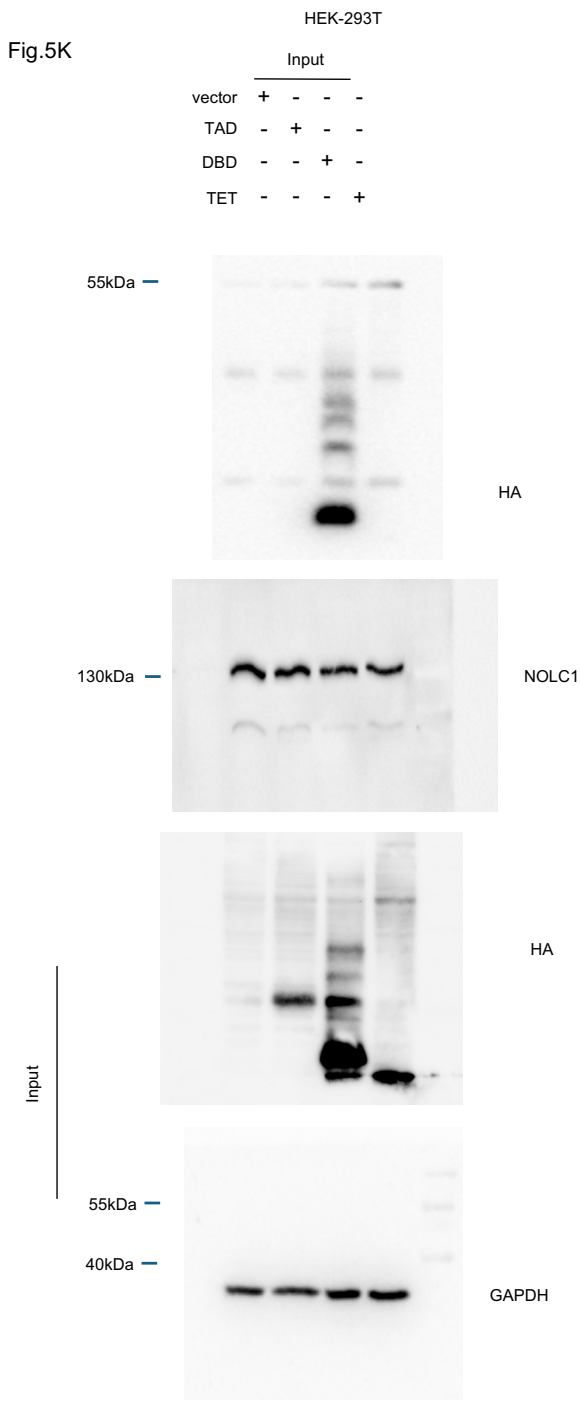

Fig.5L

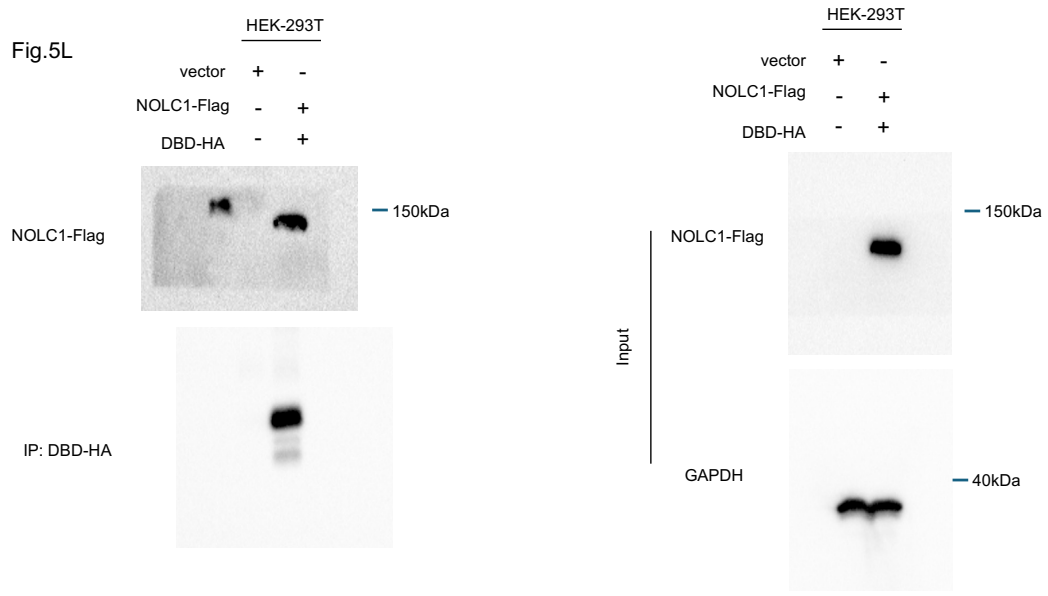

Supplement: Figure 5—source data 1. [file elife-103904-fig5-data1.zip › fig5-source data1.pdf]

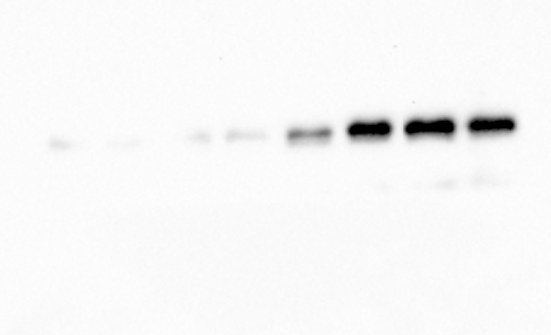

Supplement: Figure 5—source data 2. [file elife-103904-fig5-data2.zip › fig5 source data2/5I/p53 short exposure.jpg]

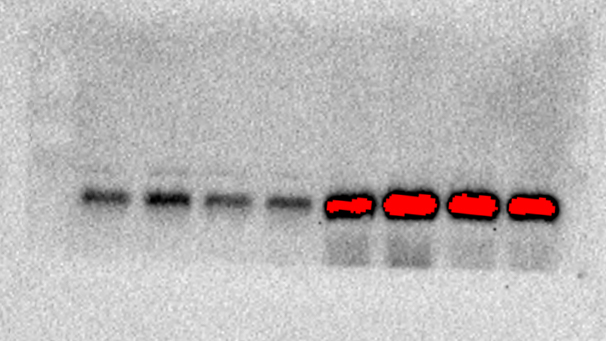

Supplement: Figure 5—source data 2. [file elife-103904-fig5-data2.zip › fig5 source data2/5I/p53 long exporsure.jpg]

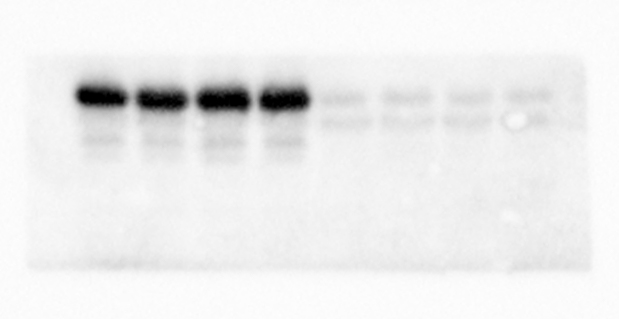

Supplement: Figure 5—source data 2. [file elife-103904-fig5-data2.zip › fig5 source data2/5I/GAPDH.2.jpg]

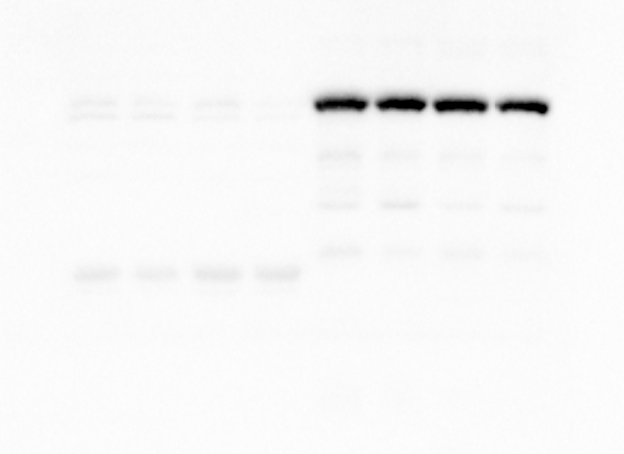

Supplement: Figure 5—source data 2. [file elife-103904-fig5-data2.zip › fig5 source data2/5I/LAMIN B1.jpg]

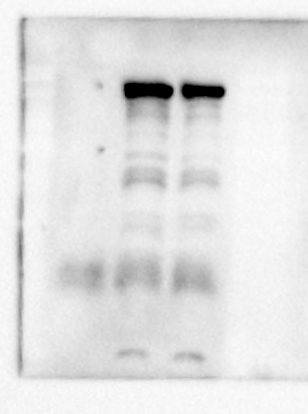

Supplement: Figure 5—source data 2. [file elife-103904-fig5-data2.zip › fig5 source data2/5G/flag.jpg]

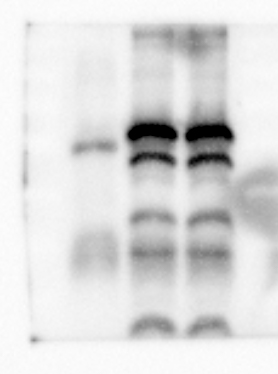

Supplement: Figure 5—source data 2. [file elife-103904-fig5-data2.zip › fig5 source data2/5G/input-HA-p53.jpg]

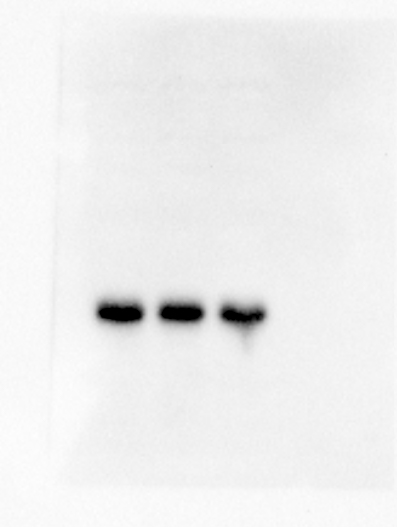

Supplement: Figure 5—source data 2. [file elife-103904-fig5-data2.zip › fig5 source data2/5G/GAP.jpg]

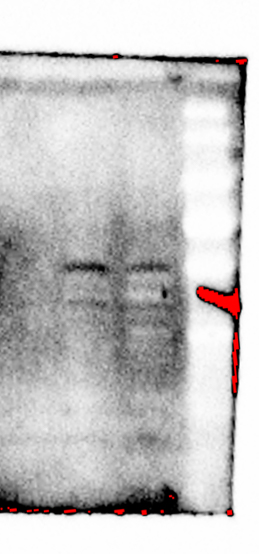

Supplement: Figure 5—source data 2. [file elife-103904-fig5-data2.zip › fig5 source data2/5G/co-ip=ha-p53.jpg]

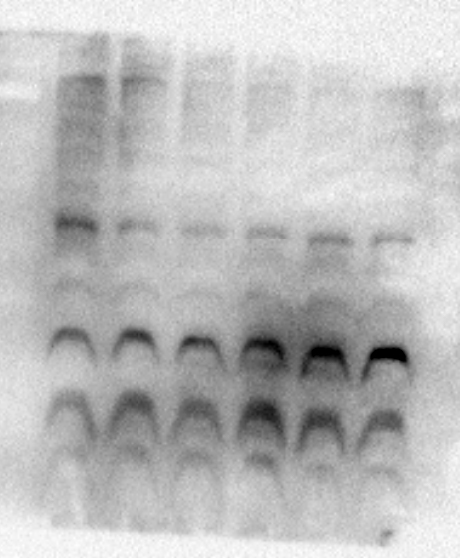

Supplement: Figure 5—source data 2. [file elife-103904-fig5-data2.zip › fig5 source data2/5A/BAX.jpg]

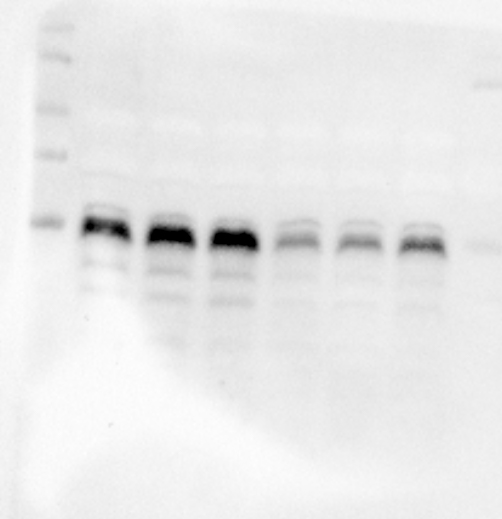

Supplement: Figure 5—source data 2. [file elife-103904-fig5-data2.zip › fig5 source data2/5A/P53.jpg]

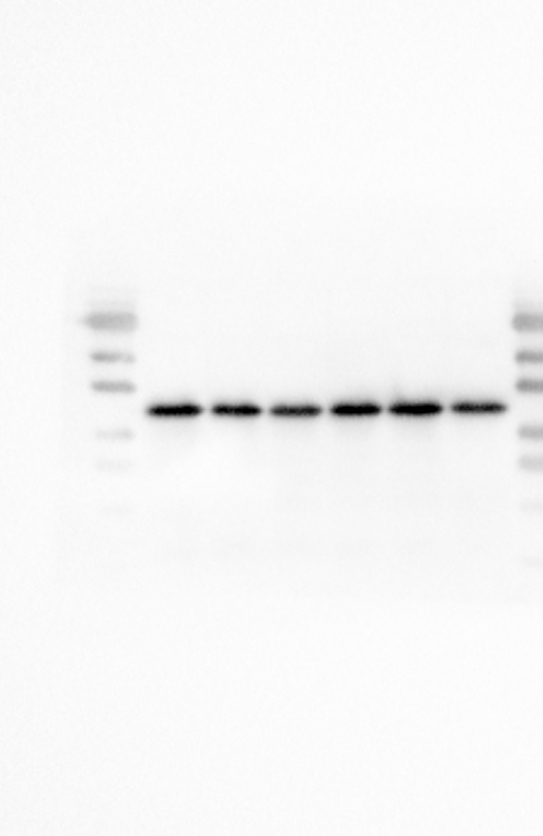

Supplement: Figure 5—source data 2. [file elife-103904-fig5-data2.zip › fig5 source data2/5A/GAPDH.jpg]

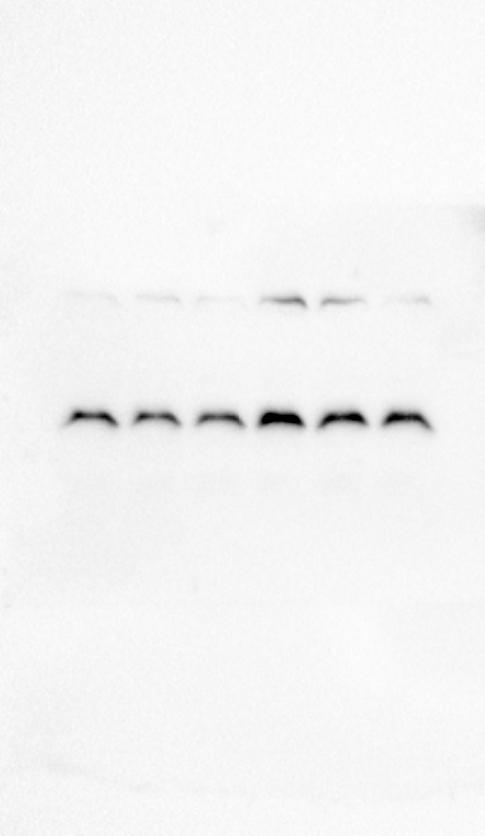

Supplement: Figure 5—source data 2. [file elife-103904-fig5-data2.zip › fig5 source data2/5A/BCL2.jpg]

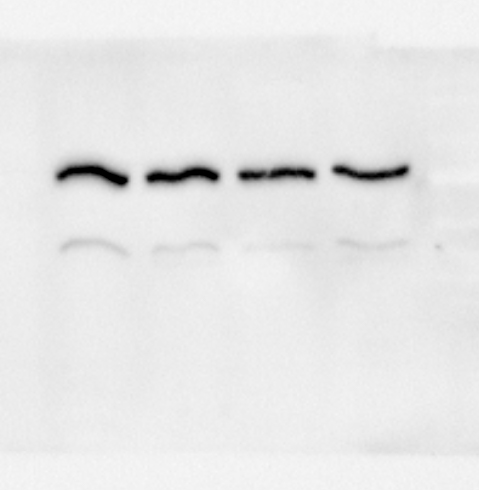

Supplement: Figure 5—source data 2. [file elife-103904-fig5-data2.zip › fig5 source data2/5k/flag-NOLC.jpg]

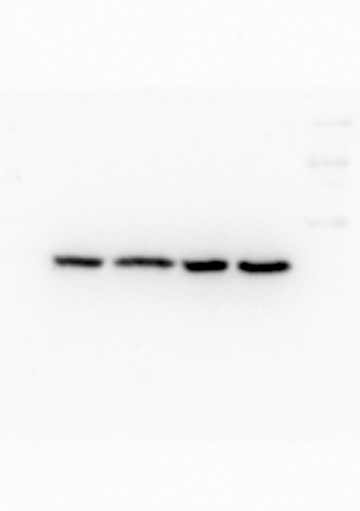

Supplement: Figure 5—source data 2. [file elife-103904-fig5-data2.zip › fig5 source data2/5k/input-GAPDH.jpg]

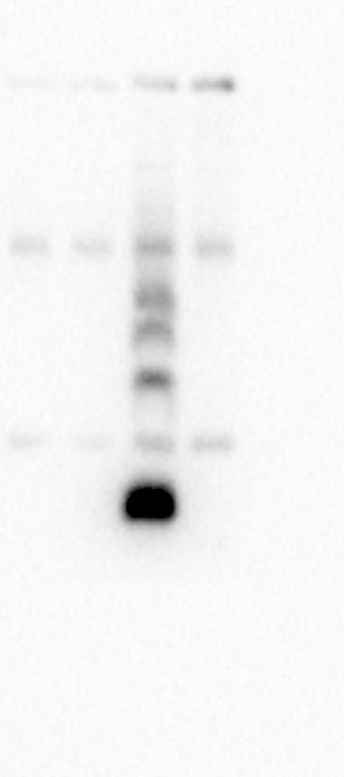

Supplement: Figure 5—source data 2. [file elife-103904-fig5-data2.zip › fig5 source data2/5k/COIP-DBD.jpg]

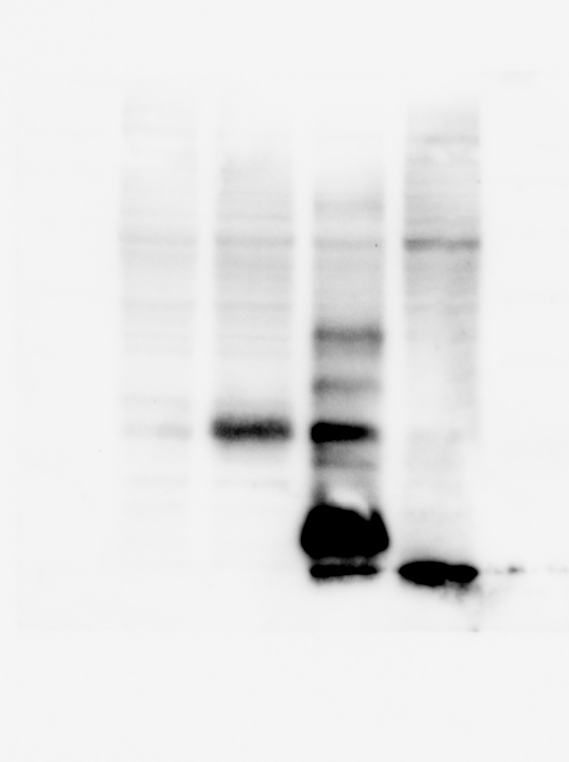

Supplement: Figure 5—source data 2. [file elife-103904-fig5-data2.zip › fig5 source data2/5k/input.jpg]

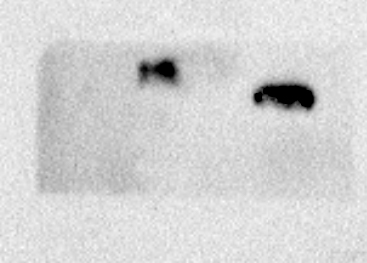

Supplement: Figure 5—source data 2. [file elife-103904-fig5-data2.zip › fig5 source data2/5L/IB∩╝Ünolc1-flag.jpg]

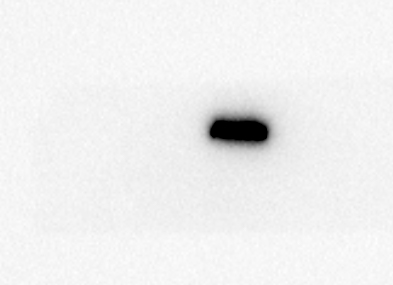

Supplement: Figure 5—source data 2. [file elife-103904-fig5-data2.zip › fig5 source data2/5L/input∩╝Ünolc1-flag.jpg]

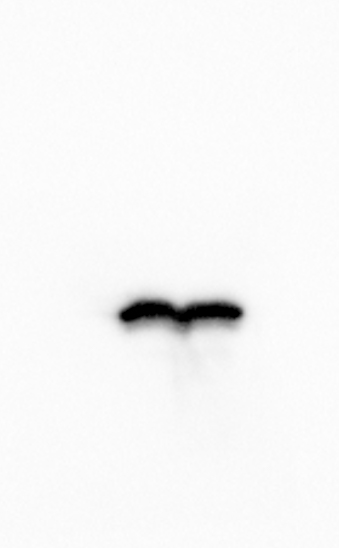

Supplement: Figure 5—source data 2. [file elife-103904-fig5-data2.zip › fig5 source data2/5L/input∩╝ÜGAPDH.jpg]

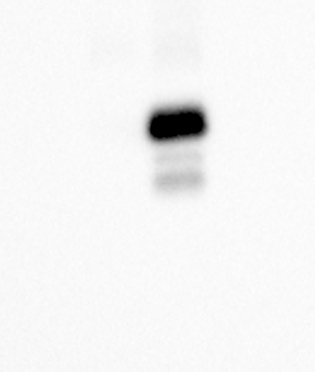

Supplement: Figure 5—source data 2. [file elife-103904-fig5-data2.zip › fig5 source data2/5L/IP∩╝ÜHA-DBD.jpg]

Fig.6C

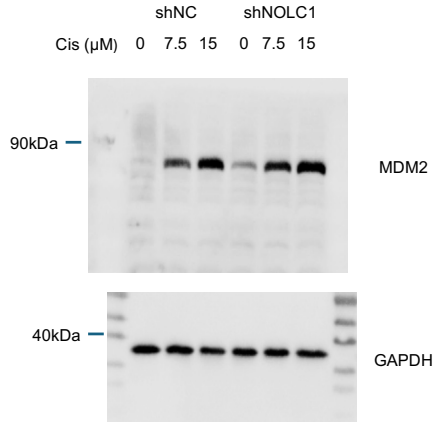

Fig.6G

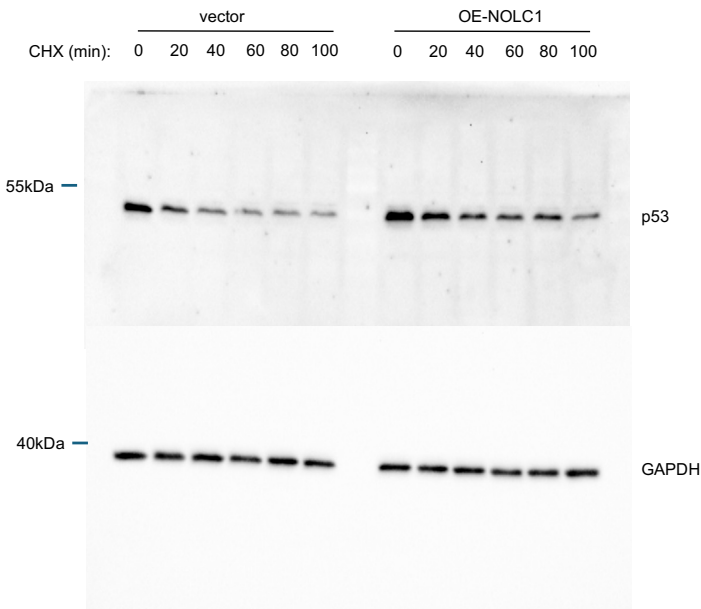

Fig. 6H

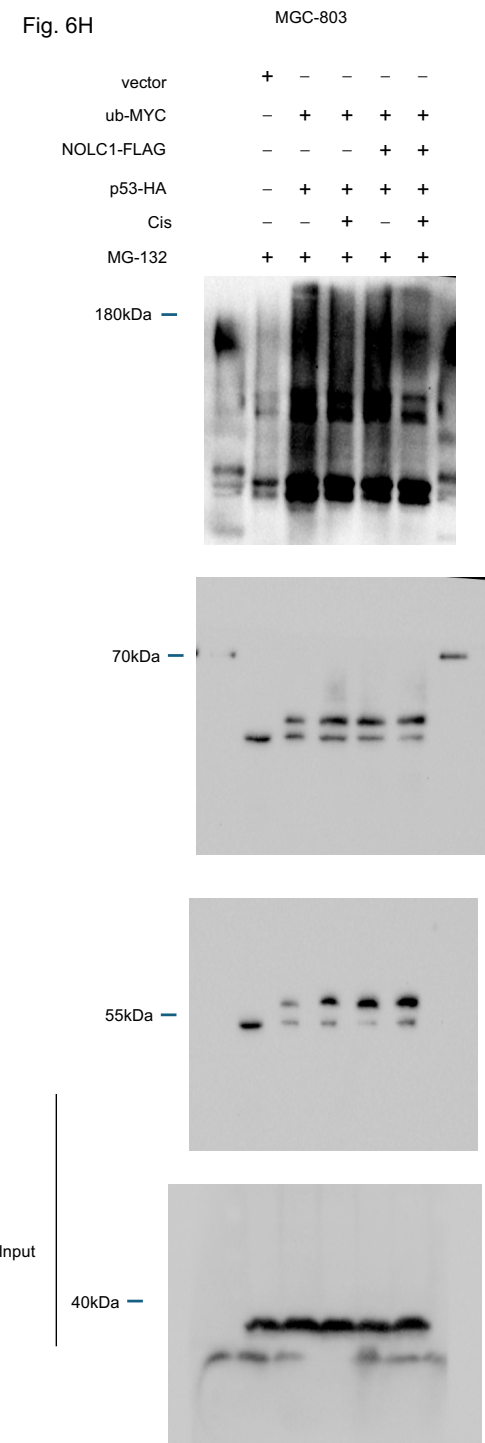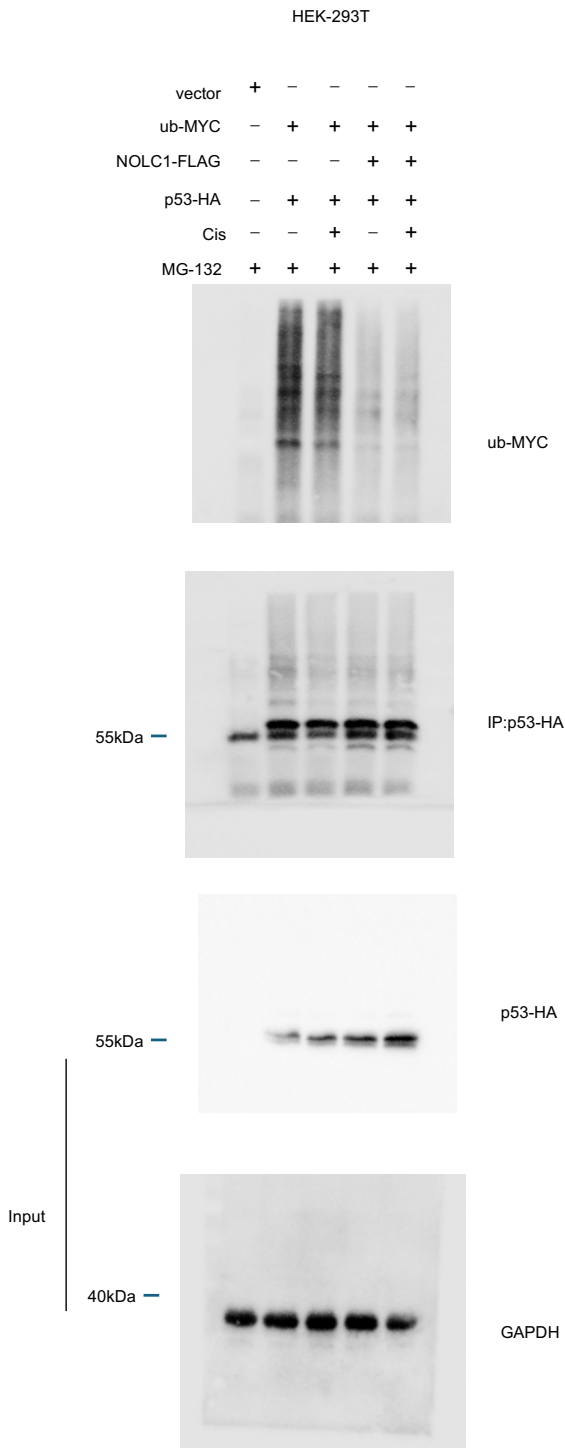

Supplement: Figure 6—source data 1. [file elife-103904-fig6-data1.pdf]

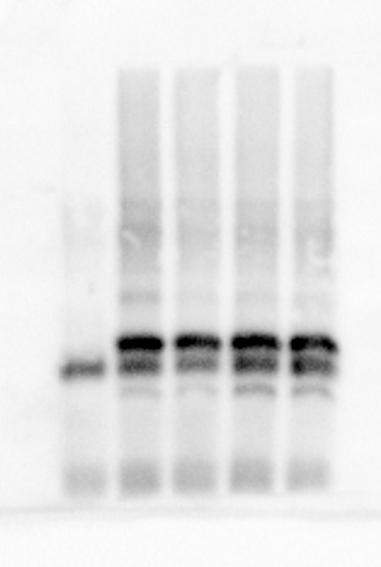

Supplement: Figure 6—source data 2. [file elife-103904-fig6-data2.zip › fig6 source data2/6H/IP ha-293t.jpg]

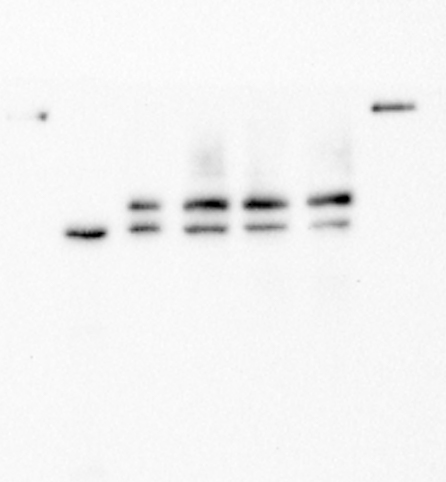

Supplement: Figure 6—source data 2. [file elife-103904-fig6-data2.zip › fig6 source data2/6H/ha-2.jpg]

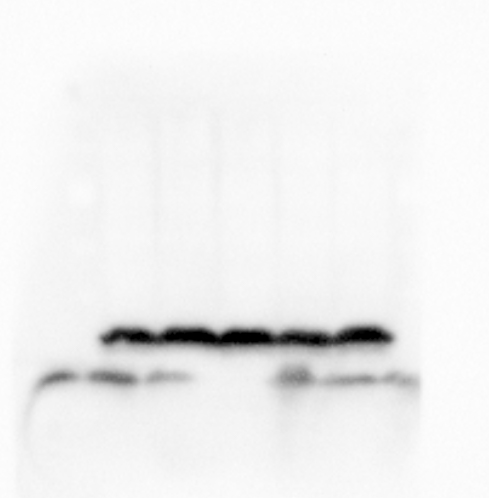

Supplement: Figure 6—source data 2. [file elife-103904-fig6-data2.zip › fig6 source data2/6H/input-GAPDH.jpg]

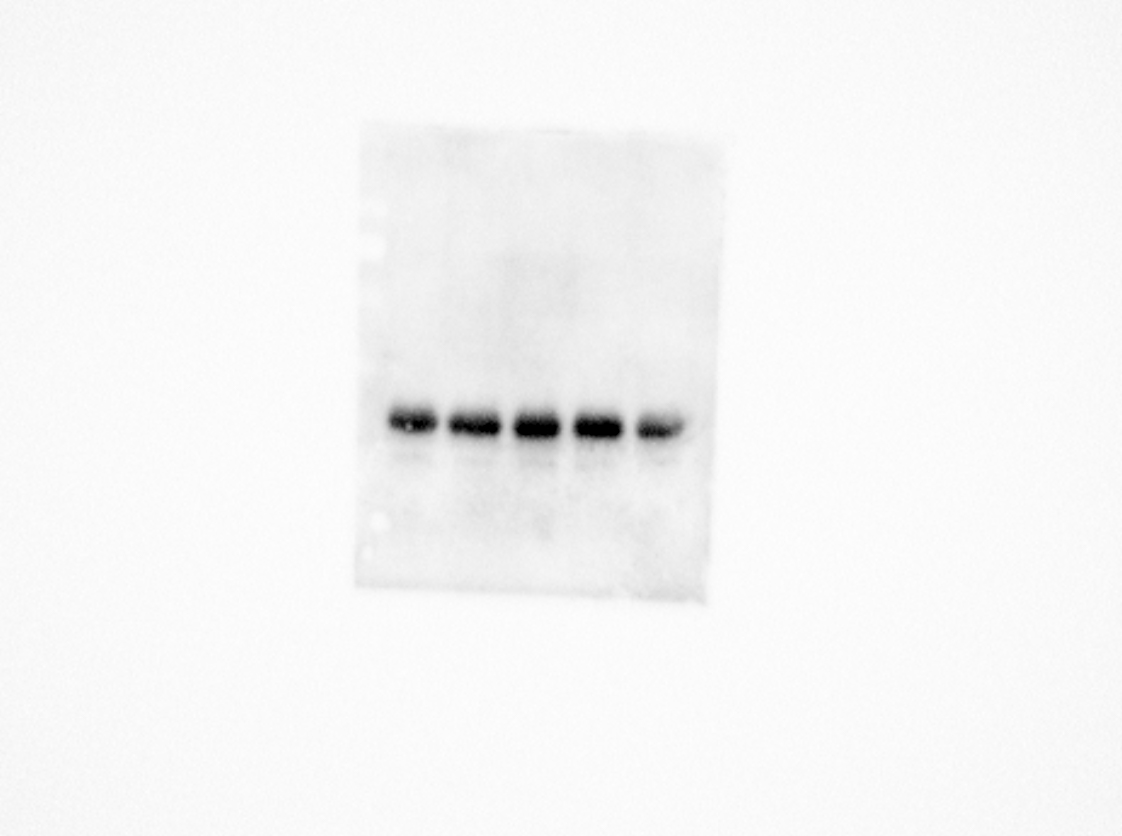

Supplement: Figure 6—source data 2. [file elife-103904-fig6-data2.zip › fig6 source data2/6H/INPUT-GAPDH-293t.jpg]

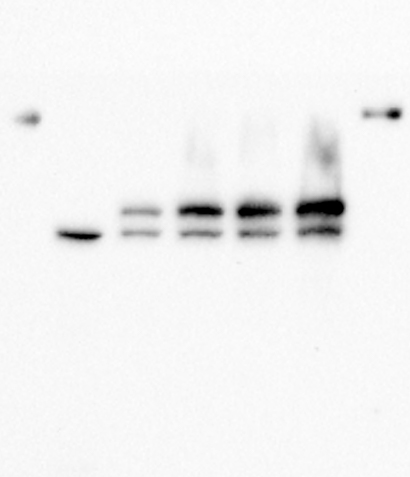

Supplement: Figure 6—source data 2. [file elife-103904-fig6-data2.zip › fig6 source data2/6H/input-HA-τô╢3.jpg]

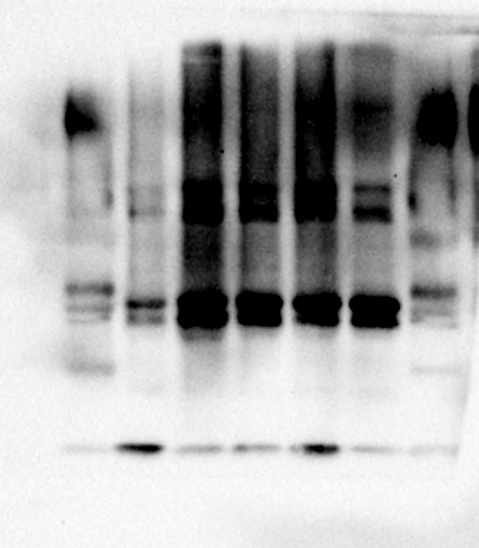

Supplement: Figure 6—source data 2. [file elife-103904-fig6-data2.zip › fig6 source data2/6H/ub-myc.jpg]

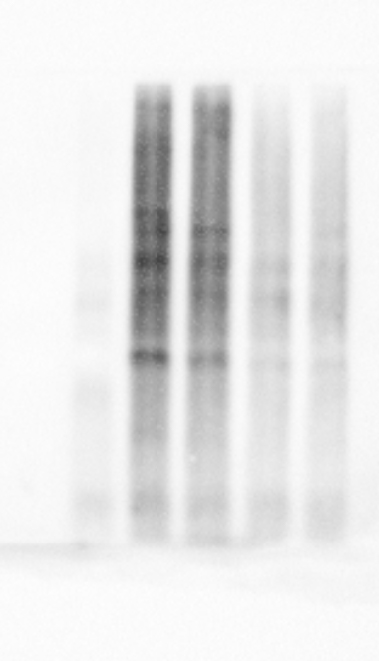

Supplement: Figure 6—source data 2. [file elife-103904-fig6-data2.zip › fig6 source data2/6H/ub-myc-293t.jpg]

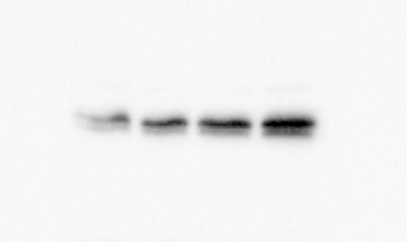

Supplement: Figure 6—source data 2. [file elife-103904-fig6-data2.zip › fig6 source data2/6H/p53-HA-input.jpg]

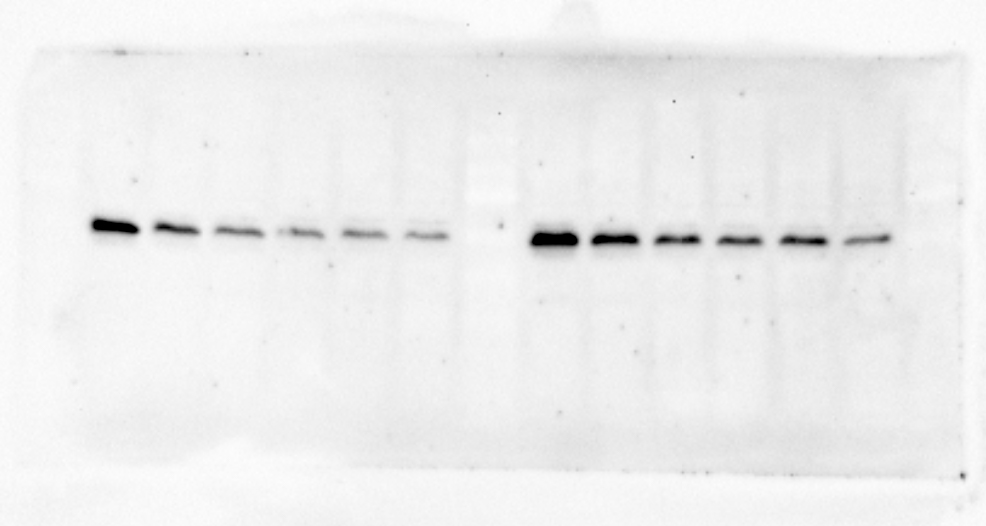

Supplement: Figure 6—source data 2. [file elife-103904-fig6-data2.zip › fig6 source data2/6G/CHX-p53.jpg]

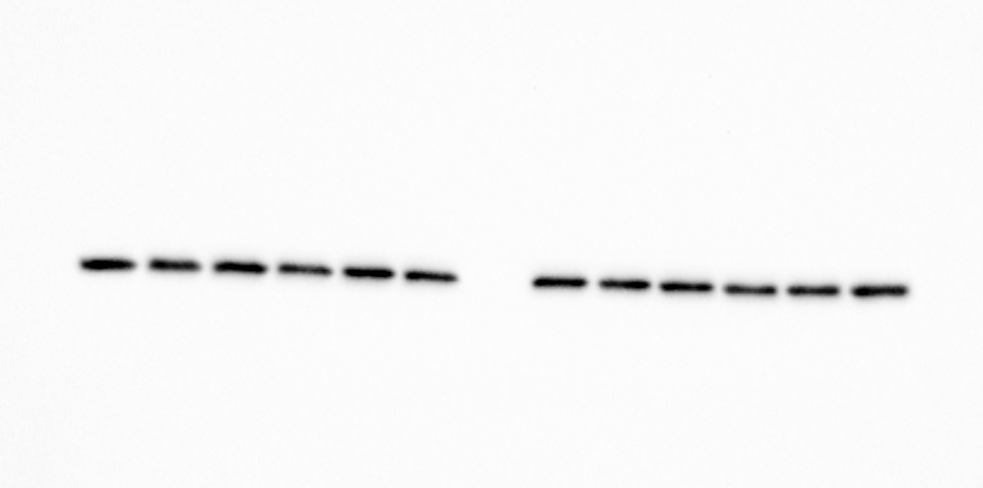

Supplement: Figure 6—source data 2. [file elife-103904-fig6-data2.zip › fig6 source data2/6G/CHX-GAP.jpg]

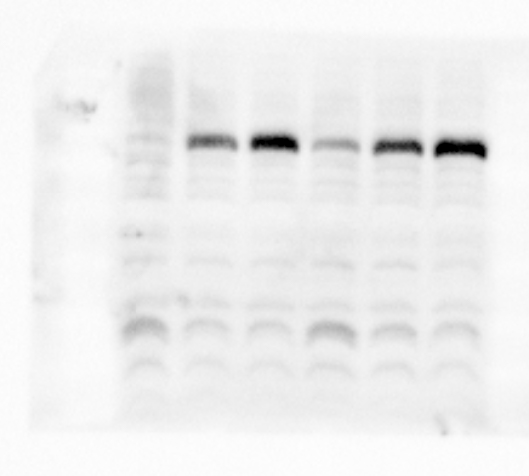

Supplement: Figure 6—source data 2. [file elife-103904-fig6-data2.zip › fig6 source data2/6C/mdm2-σà¿τ╗åΦâ₧.3.jpg]

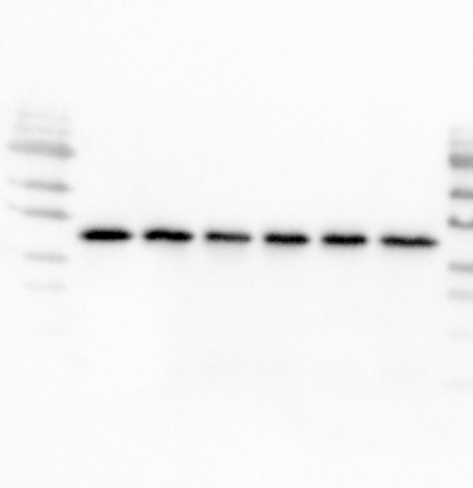

Supplement: Figure 6—source data 2. [file elife-103904-fig6-data2.zip › fig6 source data2/6C/gapdh.jpg]

Figure 6-figure supplement 1D

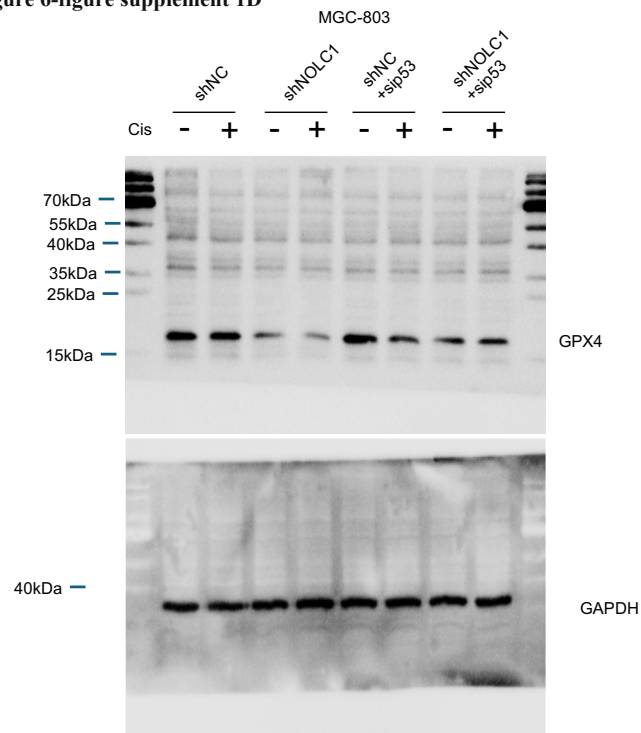

Supplement: Figure 6—figure supplement 1—source data 1. [file elife-103904-fig6-figsupp1-data1.pdf]

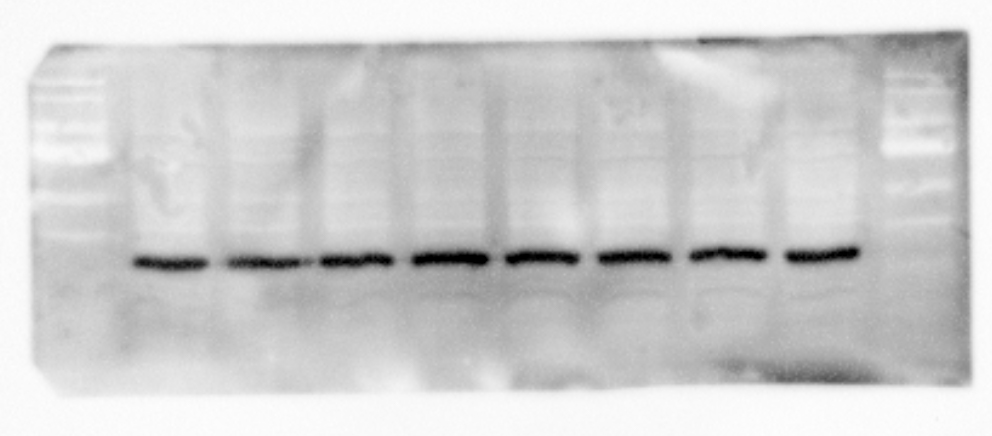

Supplement: Figure 6—figure supplement 1—source data 2. [file elife-103904-fig6-figsupp1-data2.zip › fig6 fig supplement 1source data2/GAPDH.jpg]

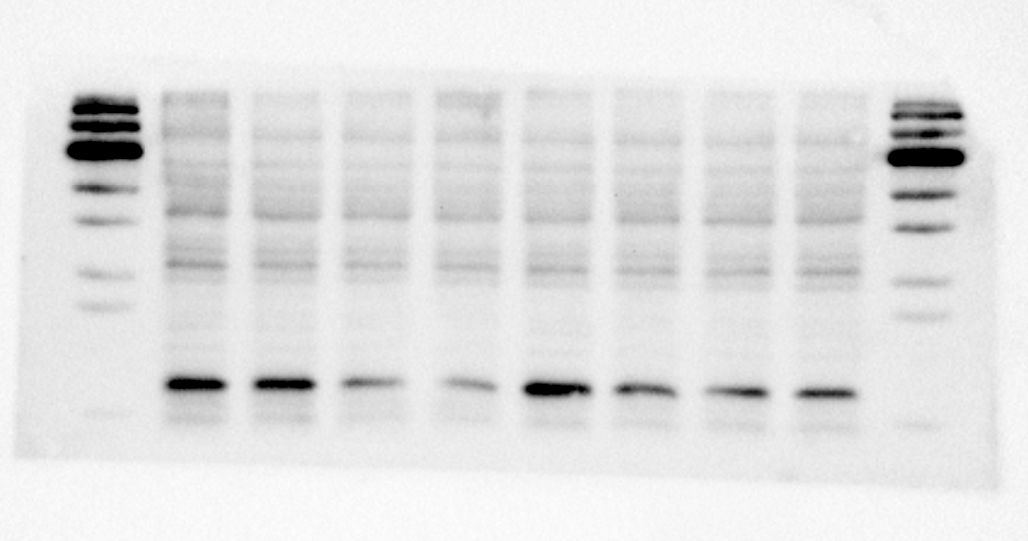

Supplement: Figure 6—figure supplement 1—source data 2. [file elife-103904-fig6-figsupp1-data2.zip › fig6 fig supplement 1source data2/gpx4.jpg]
